# Tenovin 3 induces apoptosis and ferroptosis in EGFR 19del non small cell lung cancer cells

**DOI:** 10.1038/s41598-024-58499-5

**Published:** 2024-04-01

**Authors:** Sha Lv, Qianrong Pan, Weijin Lu, Weisong Zhang, Naike Wang, Lijuan Huang, Lianjing Li, Jieyao Liu, Jiamei Ma, Zhan Li, Yong Huang, Qiudi Deng, Xueping Lei

**Affiliations:** 1 The Fifth Affiliated Hospital,Guangdong Province & NMPA & State Key Laboratory,School of Pharmaceutical Sciences, Guangzhou Medical University, Guangzhou, 511436 People’s Republic of China; 2 The Fifth Affiliated Hospital of Jinan University (Heyuan Shenhe People’s Hospital), Heyuan, 517000 China; 3 GMU-GIBH Joint School of Life Sciences, The Guangdong-Hong Kong-Macau Joint Laboratory for Cell Fate Regulation and Diseases, Guangzhou Medical University, Guangzhou, 511436 People’s Republic of China; 4 Medicine and Health Science College, Guangzhou Huashang College, Guangzhou, People’s Republic of China

**Keywords:** Non-small cell lung cancer (NSCLC), Epidermal growth factor receptor (EGFR) mutation, Tenovin-3, Apoptosis, Ferroptosis, Drug discovery, Molecular medicine, Oncology

## Abstract

Epidermal growth factor receptor (EGFR) exon 19 deletion is a major driver for the drug resistance of non-small cell lung cancer (NSCLC). Identification small inhibitor capable of selectively inhibiting EGFR-19del NSCLC is a desirable strategy to overcome drug resistance in NSCLC. This study aims to screen an inhibitor for EGFR exon 19 deletion cells and explore its underlying mechanism. High through-put screen was conducted to identify an inhibitor for EGFR-19del NSCLC cells. And tenovin-3 was identified as a selective inhibitor of PC9 cells, an EGFR-19del NSCLC cells. Tenovin-3 showed particular inhibition effect on PC9 cells proliferation through inducing apoptosis and ferroptosis. Mechanistically, tenovin-3 might induce the apoptosis and ferroptosis of PC9 cells through mitochondrial pathway, as indicated by the change of VDAC1 and cytochrome c (cyt c). And bioinformatics analyses showed that the expression levels of SLC7A11 and CPX4 were correlated with NSCLC patient’s survival. Our findings provide evidences for tenovin-3 to be developed into a novel candidate agent for NSCLC with EGFR exon 19 deletion. Our study also suggests that inducing ferroptosis may be a therapeutic strategy for NSCLC with EGFR exon 19 deletion.

## Introduction

Non-small cell lung cancer (NSCLC) is one of most aggressive cancers with high recurrence and mortality. Although tremendous efforts have been made to develop effective therapy strategies for NSCLC, the overall survival of patient is still far from satisfactory^1,2^. The mutation of epidermal growth factor receptor (EGFR) accounts for majority of the metastasis and relapse of NSCLC^3^. EGFR exon 19 deletions is one of most common EGFR mutations, with a prevalence of approximately 44% ^4,5^. However, the emergence of acquired drug resistance seriously hampers the application of these inhibitors^6,7^. Therefore, it is of great clinical significance to develop specific and effective agents for NSCLC patient with EGFR exon 19 deletions.

Ferroptosis is a non-apoptotic type of cell death that was identified in 2012. It is distinct from other types of cell death and it is characterized with iron-based lipid peroxidation accumulation. During ferroptosis, cells are characterized with many ultra-micromorphological features such as shrinkage of mitochondrial membrane, smaller mitochondrial size and less mitochondrial ridges. Mechanistically, ferroptosis is triggered by complex and abnormal biochemical processes including iron overload, exhaustion of glutathione (GSH), co-enzyme Q10^8,9^. Solute carrier family seven member 11(SLC7A11/xCT), a unit of system x c^−^, and Glutathione peroxidase 4 (GPX4) are two critical regulators of ferroptosis^10^. Emerging studies have recognized ferroptosis-dependent cell death as a novel therapeutic strategy for cancer. And many agents that can induce ferroptosis have developed and applied for cancer therapy, such as RSL3 and Erastin^11,12^. Recently, several studies suggest that inducing ferroptosis in therapy-resistant cancer cells is a promoting therapeutic strategy to trigger cell death. These cells resistant to traditional therapies are more susceptible to RSL3 when compared with those non-resistant cells in melanoma, prostate cancer, and sarcomas^13,14^.Inhibition of GPX4 significantly enhanced the anti-tumor effect of lapatininb via inducing ferroptosis in lapatinib-resistant lung cancer cells^15^. Whereas, whether inducing ferroptosis can trigger cell death in EGFR-19del cells is still unclear.

**Figure 1:**
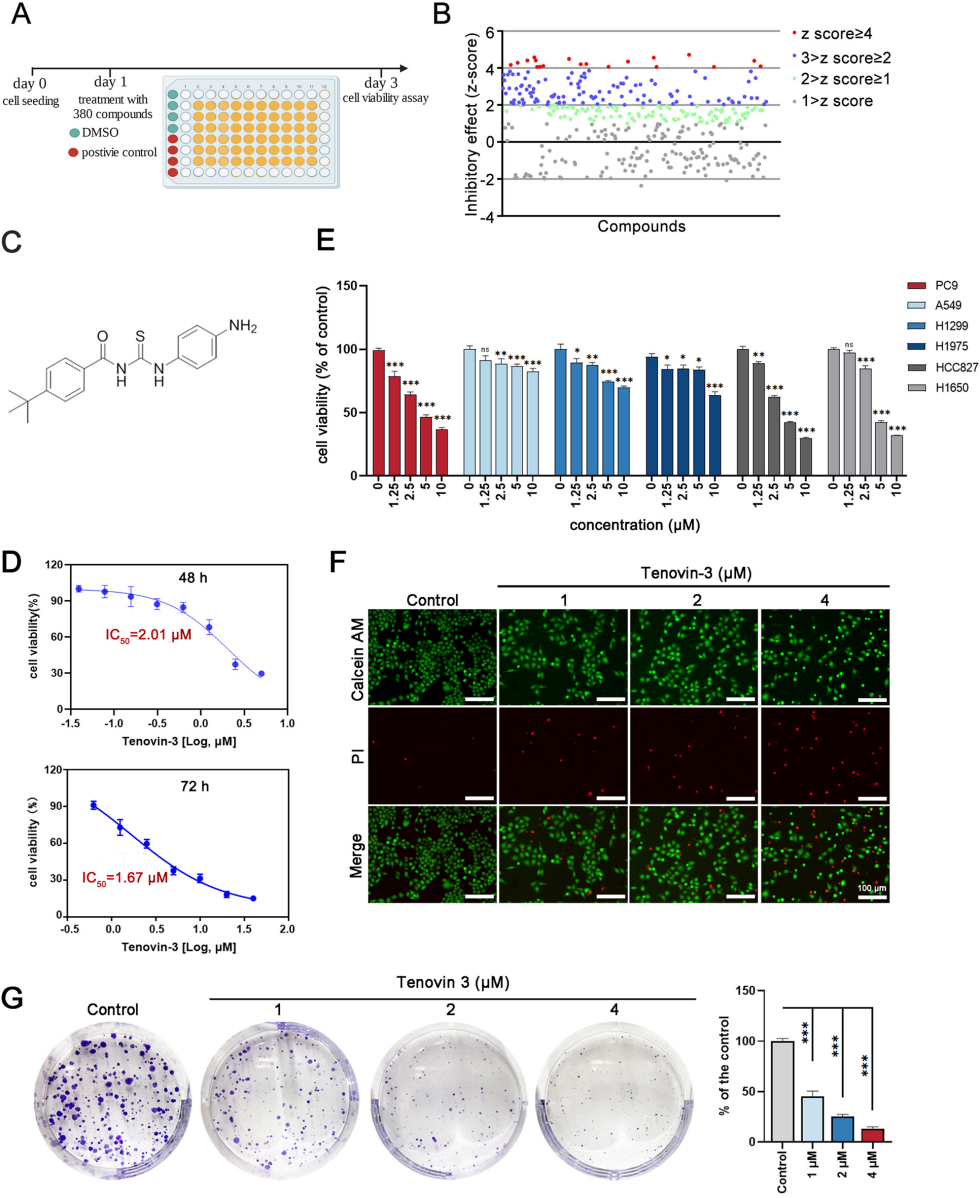
Tenovin-3 inhibits PC9 cell proliferation and colony formation. (**A**) Schematic overview of high-throughput drug screen for inhibitor of PC9 cells proliferation. (**B**) The z-scores of 380 compounds mediated inhibitory effect on the PC 9 cells proliferation. (**C**) The chemical structure of tenovin-3. (**D**) Tenovin-3 suppressed PC 9 cells proliferation. The PC9 cells were exposure with different concentrations of tenovin-3 for 48 h or 72 h, and the cell viability was detected by CCK-8 assay. (**E**) The effect of tenovin-3 on the proliferation of different NSCLC cells. The PC9, NCI-H1299, A549, NCI-H1975, NCI-H88827 and NCI-H1650 cells were treated with various concentration of tenovin-3 for 48 h. CCK-8 assay was used to detect cell viability of these cells. (**F**) Tenovin-3 induced PC9 cells death detected by Calcein-AM/ PI staining assay. (**G**) The effect of tenovin-3 on the colony formation of PC9 cells. The data are presented as mean ± SD, n = 3. ^***^P < 0.001 compared with the control group.

Recently, targeting based high-throughput screen of drugs have been developed and contributes to identify novel therapeutic agents ^16^. In the present study, we screened an inhibitor for PC9 cells (an EGFR-19del NSCLC cell lines) using high-throughput screen from an epigenetic compound library. We identified tenovin-3 (Fig. 1a) as a novel therapeutic agent against PC9 cells. Tenovin-3 showed selective inhibitory effect against PC9 cells. Further research showed that tenovin-3 obviously induced the apoptosis and ferroptosis of PC9 cells. Mechanism study revealed that tenovin-3-mediated promotion effect on apoptosis and ferroptosis might associate with the mitochondrial pathway. In addition, bioinformatics analyses proved that SLC7A11 and CPX4 expression are associated with NSCLC patient prognosis. Taken together, our data suggest that tenovin-3 is a potential candidate agent for the NSCLC patient with EGFR exon 19 deletion. Our study also indicates that inducing ferroptosis may be a potential therapeutic strategy for EGFR exon 19 deletion NSCLC.

## Results

### Tenovin-3 inhibits the proliferation and colony formation of PC9 cells

Firstly, we screened promising compounds that can suppress PC9 cells proliferation from a compound library containing 380 compounds at the concentration of 10 µM (Fig. 1A). And 18 compounds that with high inhibition effect on PC9 cells (Z-score > 4) were selected based on the Venn diagram (Fig. 1B and Supporting table 1). Among them, tenovin-3 showed the most obvious inhibition effect on PC9 cells proliferation, with an IC_50_ of 2.01 µM and 1.67 µM at 48 h and 72 h, respectively (Fig. 1C,D). And this effect was almost similar with the effect of Osimertinib on PC9 cells (IC_50_ is 1.86 µM, 48 h) (Supporting Fig. 1A). Interestingly, we found that tenovin-3 showed selective inhibition effect on the proliferation of PC9 cells. The effect of tenovin-3 on EGFR wide type cells NCI-H1299 cells and A549 cells were slight. And its effect on PC9 cells was better than NCI-H1975 (L858R EGFR mutation), NCI-H1650 and NCI-H8827 (E746-A750 EGFR mutation) cells (Fig. 1E). In addition, its inhibition effect on PC9 cells was also better than that on NHBE and BEAS-2B cells (two Human normal lung bronchial epithelial cells) (Supporting Fig. 1). And then, 1 μM, 2 μM and 4 μM of tenovin-3 were selected for further experiments. And the control group was treated with 0.02% dimethyl sulphoxide (DMSO), which is similar with the DMSO concentration in 4 μM tenovin-3 group. Calcein-AM/PI staining assay was conducted to further confirm the effect of tenovin-3. The results showed that tenovin-3 markedly induced PC9 cells death in a dose-dependent manner. The green fluorescence staining living cells in tenovin-3 group was obviously reduced, and the red fluorescence representing dead cells was significantly increased when compared with the control cells (Fig. 1F). Similar results were observed in colony formation assay. Tenovin-3 significantly reduced the number of colonies in a dose-dependent manner, the colonies numbers were 47.13%, 26.92% and 14.21% of the control group in 1 μM, 2 μM and 4 μM groups, respectively (Fig. 1G). All these results showed that tenovin-3 suppressed the proliferation and colony formation of PC9 cells.

### Tenovin-3 induces PC9 cells apoptosis

**Figure 2:**
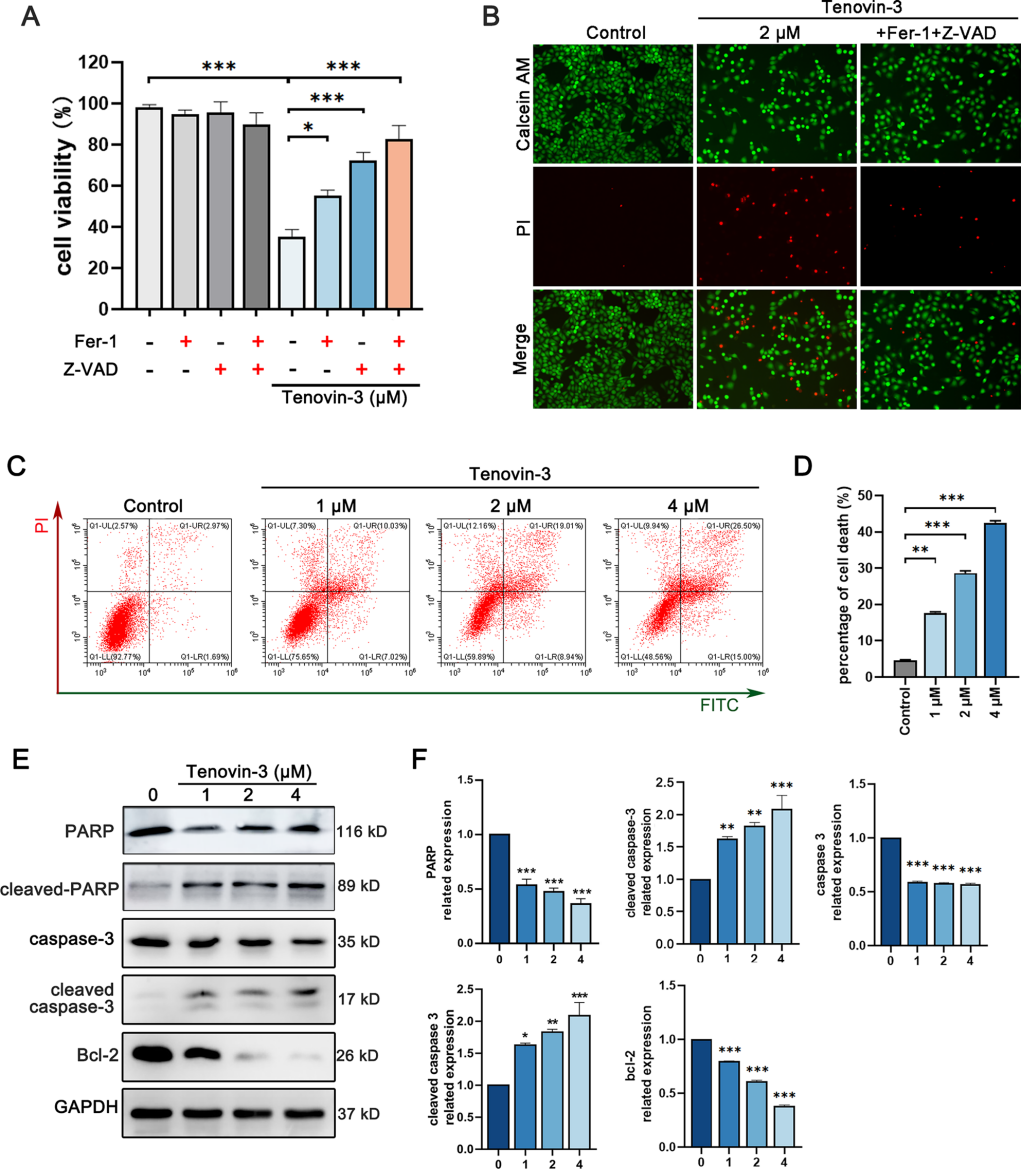
Tenovin-3 induces PC9 cells apoptosis. (**A**-**B**) The PC9 cells were co-treated with tenovin-3, apoptosis inhibitor Z-VAD-FMK or ferroptosis inhibitor Fer-1, and the cell viability was detected by CCK-8 assay (**A**) and Calcein-AM/ PI staining assay (**B**). (**C**-**D**) Tenovin-3 induced PC9 cells apoptosis indicated by flow cytometer assay. PC9 cells were exposure to various concentrations of tenovin-3 for 48 h, and then Annexin V/PI assay was used to detect apoptosis rate. The representative images and statistical data were displayed in (**C**) and (**D**). (**E**–**F**) The effect of tenovin-3 on the expression of apoptosis related protein detected by Western blotting. PC9 cells were treated with various concentrations of tenovin-3, and the cells were collected and subjected for Western blotting. GAPDH was set as a loading control. The representative blots and statistical data were presented in (**C**) and (**D**). The blots were cut and then incubated with antibodies. The uncropped version of the western blots is displayed in Supplementary Fig. 8. The data are presented as mean ± SD, n = 3. ^*^P < 0.05, ^**^P < 0.01, ^***^P < 0.001 compared with the control group.

To further evaluate the effect of tenovin-3 on PC9 cells, the death manner was analyzed. The inhibitor of apoptosis (Z-VAD-FMK), cell autophagy inhibitor (3-methyladenine) and ferroptosis inhibitor (ferrostatin-1, Fer-1)) were used in this experiment. The results showed that tenovin-3 induced inhibition effect on PC9 cells was partly abolished by Z-VAD-FMK or Fer-1 treatment, but not affected by 3-methyladenine. Fer-1 combined with Z-VAD-FMK treatment almost abolished tenovin-3 mediated inhibition effect on PC9 cells proliferation (Fig. 2A). Calcein-AM/PI staining assay also confirmed this result, Z-VAD-FMK combined with Fer-1 treatment significantly attenuated tenovin-3 mediated inhibition effect on PC9 cells (Fig. 2B). These results suggest that tenovin-3-induced cell death of PC9 cells may through apoptosis and ferroptosis way. The Annexin V/PI staining assay was conducted to further evaluate whether tenovin-3 induce PC9 cells apoptosis. We found that tenovin-3 obviously induced PC9 cell apoptosis in a dose-dependent manner (Fig. 2C,D). Furthermore, Western blotting verified that tenovine-3 induced the cleavage of PARP and caspase3, along with Bcl-2 (an anti-apoptotic protein) expression level decrease (Fig. 2E,F). These results indicated that tenovin-3 induced PC9 cell apoptosis.

### Tenovin-3 treatment result in PC9 cells ferroptosis

**Figure 3:**
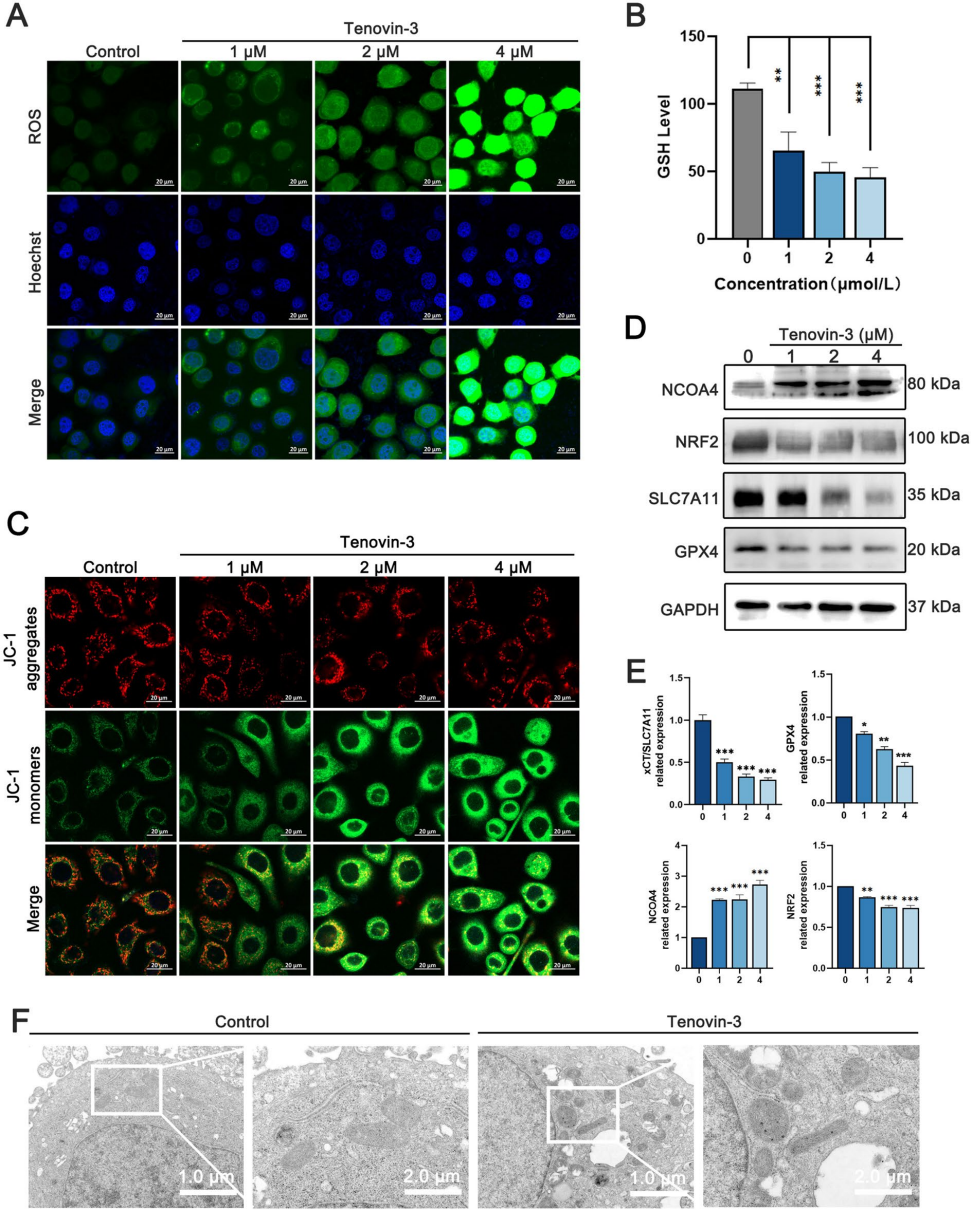
Tenovin-3 treatment induced PC9 cells ferroptosis. (**A**) The ROS level in PC9 cells with tenovin-3 treatment detected by immunofluorescence assay using DCFH-DA staining. (**B**) The level of glutathione in PC9 cells after tenovin-3 treatment. (**C**) The effect of tenovin-3 on MMP. The PC9 cells were treated with various concentrations of tenovin-3, and the MMP were detected by JC-1 staining assay. (**D**-**E**) The effect of tenovin-3 on the expression of ferroptosis regulators. The PC9 cells were treated with various concentrations of tenovin-3 for 24 h, and then the cells were collected and subjected for Western blotting. The blots were cut and then incubated with antibodies. The uncropped version of the western blots is displayed in Supplementary Fig. 8. The representative blots were presented in (**D**), and the statistical data was showed in (**E**). (**F**) The ultrastructural features of mitochondrion in PC9 cells that have been treated with various concentration of tenovin-3 were observed by TEM. The data are presented as mean ± SD, n = 3. ^*^P < 0.05 and ^***^P < 0.001 compared with the Vehicle group.

We then further investigated whether tenovin-3 induce PC9 cells ferroptosis. Immunofluorescence assay showed that the accumulation of intracellular ROS was obviously increased after tenovin-3 treatment (Fig. 3A). Further detection of glutathione, a critical characteristic of ferroptosis, showed that tenovin-3 treatment reduced glutathione level in PC9 cells in a dose-dependent manner (Fig. 3B). And MitoProbe TM JC-1 staining showed that tenovin-3 obviously reduced mitochondrial membrane potential (MMP) of PC9 cells, which is a key indicator of mitochondrial activity (Fig. 3C). And we also detected whether tenovin-3 affect the several vital regulators of ferroptosis using Western blotting. The results showed that tenovin-3 treatment effectively reduced the expression of SLC7A11, GPX4 and NRF2 in PC9 cells in a dose-dependent manner, accompanied by the up-regulation of NCOA4 (Fig. 3D,E). Additionally, Transmission electron microscope (TEM) assay was performed to observe morphological characteristics of ferroptosis. The cell membrane in the vehicle group was intact with a small number of surrounding pseudopodia and processes, whereas tenovin-3 treatment caused shrunken mitochondria, even more absence of mitochondria cristae in 4 µM tenovin-3 group (Fig. 3F). All these results proved that tenovin-3 significantly induced ferroptosis in PC9 cells.

### Tenovin-3 may induce PC9 cell apoptosis and ferroptosis through the mitochondrial pathway

**Figure 4:**
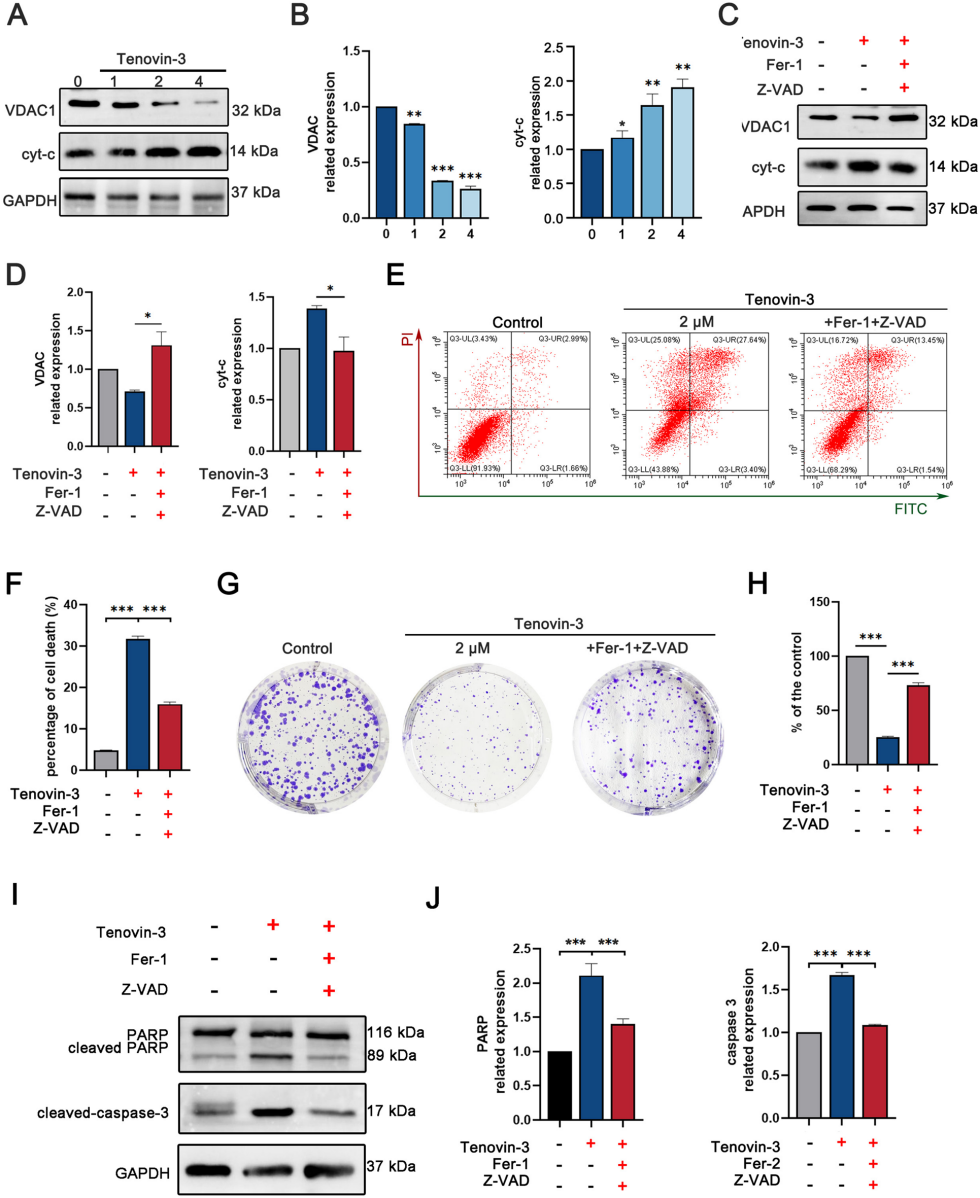
Tenovin-3 induces cell apoptosis through the mitochondrial pathway. (**A**-**B**) The effect of tenovin-3 on cyt-c and VDAC1 expressions. (**C**-**D**) Z-VAD-FMK and ferroptosis treatment weaken tenovin-3 mediated release of cyt-c and VDAC1. The PC9 cells were pre-treated with Z-VAD-FMK and ferroptosis for 6 h, and then the cells were exposure with tenovin-3 for 48 h. After that, the cells were collected and applied for Western blotting. (E–F) Z-VAD-FMK and Fer-1 treatment attenuated tenovin-3 induced effect on PC9 cells apoptosis detected by Annexin V/PI assay. (G-H) Z-VAD-FMK and Fer-1 treatment weaken tenovin-3-induced inhibition effect on PC 9 cells indicted by colony formation assay. (I-J) Z-VAD-FMK and Fer-1 treatment impeded tenovin-3 mediated effect on PC 9 cells. The blots were cut prior to incubation with antibodies. The uncropped version of the western blots is presented in Supplementary Fig. 8. The data are presented as mean ± SD, n = 3. ^*^P < 0.05, ^**^P < 0.05 and ^***^P < 0.001 compared with the control group.

Considering that mitochondrial pathway is crucial for the regulation of apoptosis and ferroptosis^17^, we detected whether tenovin-3 affect VDAC1 expression, a critical regulator of mitochondria-mediated apoptosis and ferroptosis^18,19^. The results proved that tenovin-3 treatment obviously reduced VADC1 expression, accompanied by cyt-c expression upregulation (Fig. 4A,B). And Z-VAD-FMK and Fer-1 pretreatment obviously attenuated tenovin-3 mediated inhibitory effect on VDAC1 and cyt-c expression (Fig. 4C,D). We also observed that tenovin-3 induced PC9 cells apoptosis were obviously attenuated by Z-VAD-FMK and Fer-1 treatment in Annexin V/PT assay (Fig. 4E,F). Similar results were observed in colony formation assay, tenovin-3 induced colony formation was dramatically weakened by Z-VAD-FMK combined with Fer-1 treatment (Fig. 4G,H). And the cleavage of PARP and caspase 3 were almost abolished by Z-VAD-FMK and Fer-1 treatment (Fig. 4I,J).

**Figure 5:**
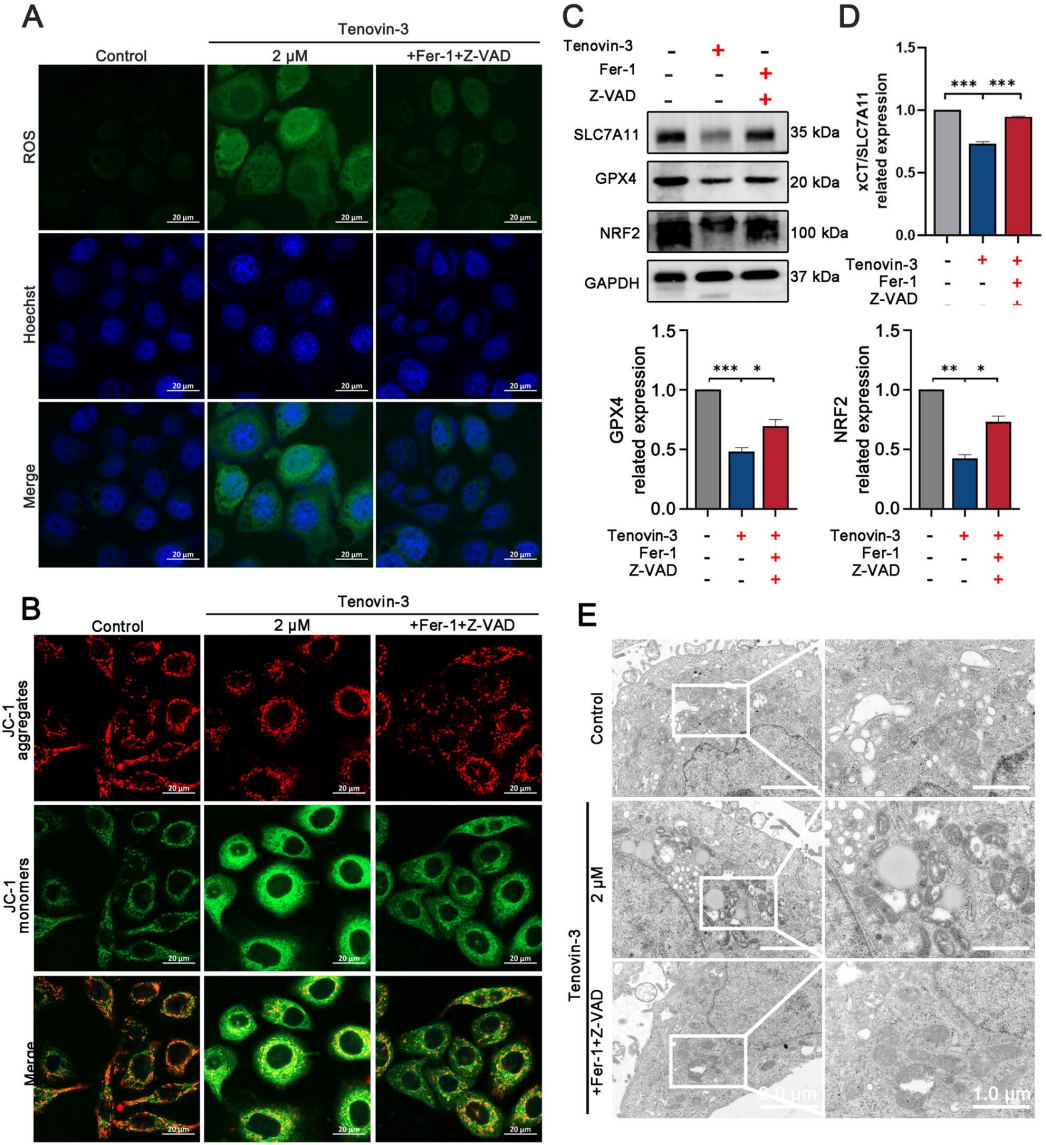
Tenovin-3 mediated cell ferroptosis might through the mitochondrial pathway. (**A**) Z-VAD-FMK and Fer-1 treatment impeded tenvin-3 induced ROS increase in PC9 cells. (**B**) Z-VAD-FMK and Fer-1 treatment attenuated tenovin-3 induced decrease of MMP. (**C**-**E**) Tenovin-3 induced PC9 ferroptosis was attenuated by Z-VAD-FMK and Fer-1 treatment indicated by Western blottin (**C**-**D**) and TEM (**E**) assays. The blots were cut and incubated with antibodies. The uncropped version of the western blots is presented in Supplementary Fig. 8. The data are presented as mean ± SD, n = 3. ^*^P < 0.05, ^**^P < 0.01, ^***^P < 0.001 compared with the control group.

Additionally, Z-VAD-FMK and Fer-1 treatment also weaken tenovin-3 mediated ROS accumulation (Fig. 5A). JC-1 staining assay confirmed that tenovin-3-mediated MMP decrease was markedly attenuated by Z-VAD-FMK and Fer-1 treatment (Fig. 5B). Western blotting demonstrated that Z-VAD-FMK and Fer-1 treatment notably attenuated tenovin-3 induced inhibition effect on SLC7A11, GPX4 and NRF2 (Fig. 5C,D). TEM assay showed that tenovin-3 induced shrunken mitochondria and decrease were significantly weaken by Z-VAD-FMK and Fer-1 treatment (Fig. 5E). All these results suggested that tenovin-3 might induce PC9 cells apoptosis and ferroptosis through the mitochondrial pathway.

### SLC7A11 and GPX4 expression are associated with the prognosis of NSCLC patients

**Figure 6:**
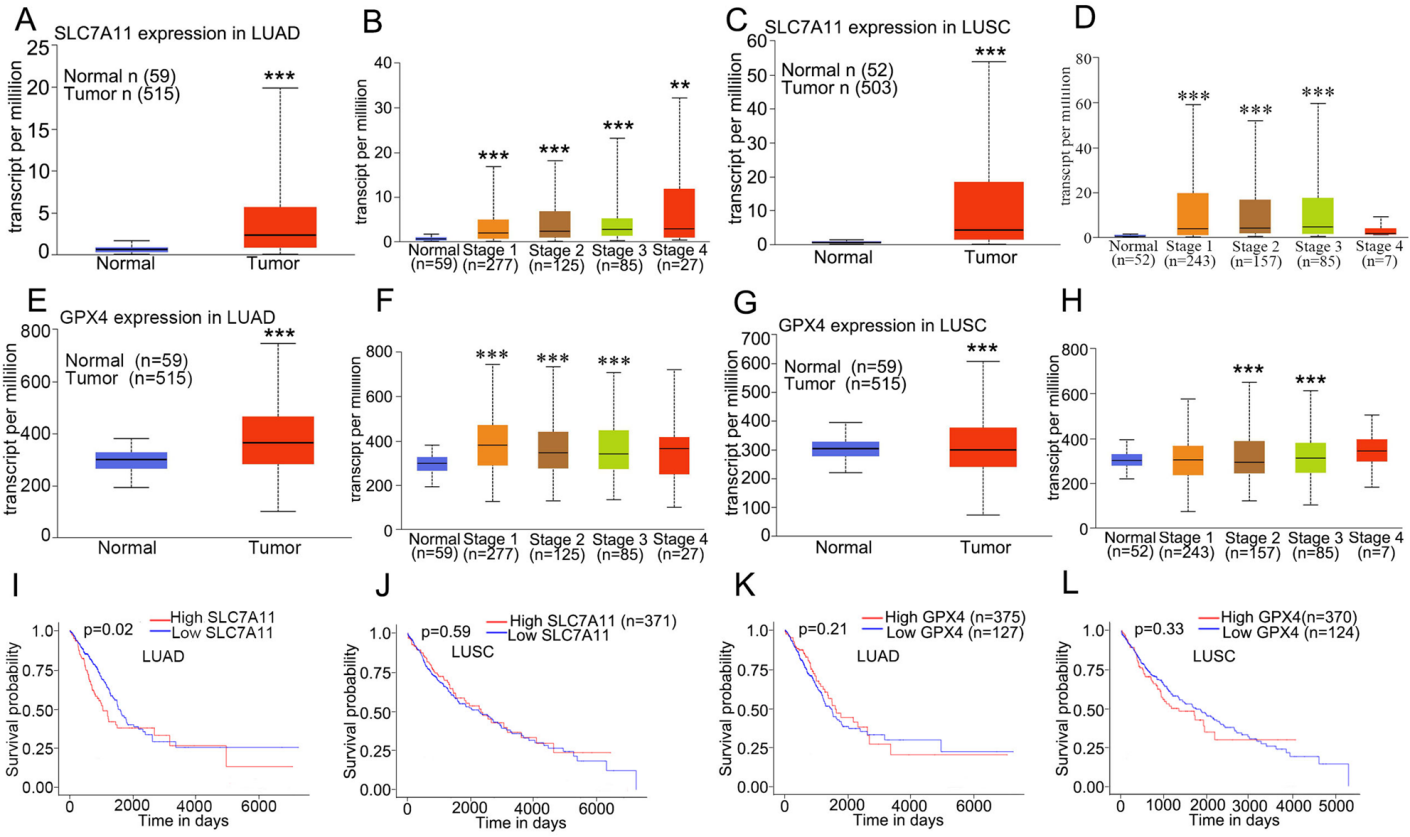
Ferroptosis may contribute to the prognosis of NSCLC patients. (**A**) The SLC7A11 expression is increased in LUAD based on TCAG platform at http://ualcan.path.uab.edu/index.html. (**B**) SLC7A11 level is correlated with the individual cancer stages of LUAD. (**C**-**D**) The GPX4 expression in LUSC tissues and its correlation with individual cancer stages of LUSC. (E–F) The GPX4 expression in LUAD (E) and its expression is related with individual cancer stage (**F**). (**G**-**H**) The GPX4 level in LUAD tissues (**G**) and it is correlated with individual cancer stage (**H**). (**I**) LUAD patients with high SLC7A11 level display a poorer survival rate than that with low SLC7A11 expression. (**J**) There is no significant correlation between SLC7A11 expression and survival of LUSC patient. (**K**-**L**) There is no significant correlation between GPX4 expression and survival of LUAD and LUSC patients. ^**^P < 0.01, ^***^P < 0.001 compared with the normal tissues.

To better clarify the possible association between ferroptosis and survival status of NSCLC patient, we conducted bioinformatics analyses using the TCGA platform (http://ualcan.path.uab.edu/index.html). We found that SLC7A11 expression was obviously increased in lung adenocarcinoma (LUAD) tissues when compared with that in normal tissues (Fig. 6A). We further analyze whether SLC7A11 expression was correlated with the pathological stage. The results showed that SLC7A11 expression was positively correlated with the individual cancer stages of LUAD (Fig. 6B). Similar results were observed in lung squamous cell carcinoma (LUSC). SLC7A11 expression was increased in LUSC (Fig. 6C), and high SLC7A11 level was associated with the individual caner stages of LUSC (Fig. 6D). We also found that GPX4, another critical regulator of ferroptosis, was up-regulated in LUAD and LUSC tissues than that in normal tissues. And GPX4 expression was partly associated with the individual cancer stages of LUAD and LUSC (Fig. 6E,F,G,H). In addition, we next analyze the relationship of SLC7A11/GPX4 expression and patient survival. We found that LUAD patients with high SLC7A11 level had a poor survival than that with low SLC7A11 expression (Fig. 6I). However, there was no significant correlation between SLC7A11 expression and LUSC patients’ survival (Fig. 6J). And no significant correlation was observed between GPX4 expression and survival of LUAD or LUSC patients (Fig. 6K,L). All these results suggested that ferrptosis might contribute to the progress of NSCLC.

## Discussion

**Figure 7:**
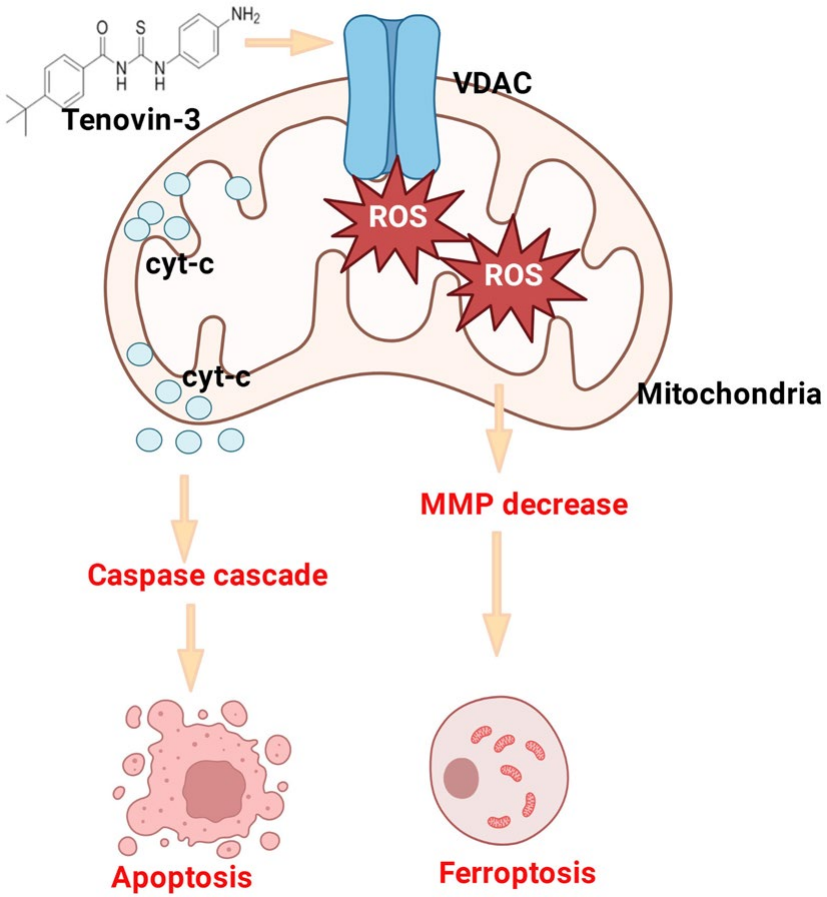
The graphic abstract of the study.

An exon 19 deletion is one of the most common EGFR mutations. Although several third-generation EGFR inhibitors including osimertinib, rociletinib and olmutinib have been developed and applied for NSCLC patients with EGFR mutation, nevertheless clinical benefits of these agents are limited in many patients. And many patients suffer from drug resistance after therapy initiation^20–22^. Thus, it is need to develop novel therapeutic strategy or drug for EGFR-19del NSCLC patients. In this study, we identified tenovin-3 as a selective inhibitor for PC9 cells (an EGFR exon 19 del cells) through high-throughput screen. We firstly found that tenovin-3 posed suppressive effect on the proliferation of PC9 cells. Further research showed that tenovin-3 induced PC9 cells apoptosis and ferroptosis through mitochondrial pathway (Fig. 7). Our study suggests that tenovin-3 is a potential anti-tumor agent for NSCLC patient with EGFR exon 19 deletion. Our study also gives a hint for exploring the anti-tumor effect and the underlying mechanism of tenovin-3.

NSCLC is one of most common malignant tumors with high morbidity and mortality^23^. EGFR mutation is a leading cause for the therapy failure of NSCLC patient. And there are approximately 40% NSCLC patient suffered from EGFR mutation in Asian^7^. EGFR mutation often increases EGFR kinase activity, leading to sustained activation of signaling pathways and continued cell proliferation. The deletion of exon 19 is one of most common EGFR mutations, accounting for approximately 40% druggable EGFR alterations^24^. Ex19 del contains large number of molecular variants including in-frame deletions, substitutions and insertions. In addition, EGFR-19del NSCLC patients often have high an incidence rate of T790M mutation after first or second generation of EGFR inhibitor therapy^5,25^. Therefore, it is of great clinical significance to search for candidate drugs targeting for EGFR-19del patients. In this study, we identified tenovin-3 as a selective inhibitor for the PC9 cells proliferation. Further research showed tenovin-3 induced the apoptosis and ferroptosis of PC9 cells through mitochondrial pathway. Besides, tenovin-3 showed obviously inhibition effect on the spheroids formed with PC9 cells and cancer associated fibroblasts (Supporting Fig. 3). All these results suggest that tenovin-3 is a potential candidate drugs for EGFR-19del NSCLC patient.

Inducing tumor cell apoptosis is a traditional therapeutic strategy for NSCLC, whereas many cancers are resistant to chemoresistant in apoptosis induction. And identifying novel agents that induces non-apoptotic cell death has been recognized as a promising therapeutic strategy for NSCLC^26,27^. Ferroptosis is a novel program cell death. It is a result of cystine depletion and massive lipid peroxidation and is critical for suppressing tumor growth^28^. And ferroptosis has been demonstrated to be involved in the acquired resistance of lapatinib, erlotinib, and vemurafenib therapy in melanoma and prostatic cancer^14,29,30^. Ferroptosis has been showed to involve in several mutations of NSCLC. Ferroptosis-protective gene such as SCD and AKR1C1/2/3 are increased in NSCLC cells with STK11/KEAP1 mutation. And these cells are resistant to GPX4 inhibition therapy. And NSCLC cells with KRAS mutation are more responsive to ferroptosis inducer^31,32^. In addition, several studies showed that ferroptosis also involved in the drug resistance of NSCLC with EGFR mutation. GPX4 level was up-regulated in lapatinib resistant A549 cells and NCI-H1994 cells, and GPX4 silencing significantly enhanced the anti-tumor effect of lapatinib^15^. Besides, xenografts constructed by EGFR mutant NSCLC cells have been proved to more sensitivity to ferroptosis treatment^33^. All these researches indicate that ferroptosis may involve in the EGFR mutation of NSCLC, and inducing ferroptosis is a potential therapy strategy for EGFR-mutated NSCLC patient. Whereas, the role of ferroptosis in NSCLC with EGFR mutation is still remains unexplored. In the present study, we proved that inducing cell ferroptosis lead to cell death of PC9 cells. Further research showed that mitochondrial pathway might contribute to the ferroptosis of PC9 cells. In addition, bioinformatics analyses showed that SLC7A11 and GPX4 expression (two critical regulators of ferroptosis) are increased in LUAD and LUSC patients and their expression were correlated with the individual cancer stages of LUAD and LUSC. LUAD patients with high SLC7A11 level displayed a lower survival than that with low SLC7A11 expression. Taken these together, our study suggests that ferroptosis is associated with the NSCLC progress and inducing ferroptosis might be a potential therapy strategy for EGFR mutant NSCLC. As for the underlying mechanism mediating ferroptosis and EGFR mutation of NSCLC, we will further explore it in the future study.

Tenovin-3 is a derivate of tenovin and it is an inhibitor of SIRT2^34,35^. It suppressed tumor cell proliferation by inhibiting protein-deacetylating activities of SIRT1 and SIRT2^34,35^. Tenovin-6, another derivate of tenovin-1, combines with metformin has been showed to induce cell apoptosis in NSCLC cells with wide type EGFR^36^. However, the effect of tenovin-3 on NSCLC cells with EGFR-19del is still unclear. In this study, we firstly proved that tenovin-3 suppressed PC9 cells prefoliation by inducing apoptosis and ferroptosis. Further research revealed tenovin-3 mediated inhibition effect on PC9 cells proliferation might associated with VDAC1 expression decrease. And VDAC1 expression in PC9 cells is relatively higher than other three cells (Supporting Fig. 4). In addition, bioinformatics analyses proved that VDAC1 expression is upregulated in LUAD and LUSC tissues. LUAD patients with high VDAC1 expression had better survival than that with low VDAC1 level. VDAC1 expression had no significant difference on LUSC patient (Supporting Figs. 6A–64D and Supporting Figs. 6I–4J). We also detected the effect of tenovin-3 on p-EGFR and SIRT2. SIRT2 expression in PC9 cells was lower than that in NCI-H1299, A549 and NCI-H1975 cells. And tenovin-3 treatment also obviously reduced p-EGFR and SITR2 level (Supporting Fig. 5). However, bioinformatics analyses showed that SIRT2 expression was decreased in LUAD and LUSC tissues. And there was no significant relation between SIRT2 expression level and LUAD or LUSC patients’ survival (Supporting Fig. 6E–H,K,L). Taken these together, we speculated tenovin-3 induced PC9 cells apoptosis and ferroptosis might by inhibiting p-EGFR and VDAC1 expression, rather than SIRT2. And we will further explore the relationship of p-EGFR and VDAC1 in the future study. In addition, we also found that tenovin-6, another derivatives of tenovin also suppressed the proliferation of PC9 cells with an IC_50_ of 2.98 μM (Supporting Fig. 7). In this regard, our study suggests that tenvoin-3 and its derivates are potential candidate agent for EGFR mutant NSCLC therapy. Our study also provides strong evidence for exploring the underlying mechanism of tenovin-3 and its derivatives mediated anti-tumor effect.

In summary, our study suggests that tenovin-3 is novel potential ferroptosis inducer for EGFR ex19 del NSCLC cells. This study also indicates that induction ferroptosis might be a potential therapeutic strategy for EGFR ex19 del NSCLC.

## Materials and methods

### Regents

Tenovin-3, Fer-1, Z-VAD-FMK, RSL-3 and Osimertinib were purchased from Topscience, Inc. (Shanghai, China). Antibodies against VDAC1, cytochrome c (cyt-c), Bcl-2, PARP, cleaved-PARP, caspase 3, cleaved-caspase 3, caspase 9, cleaved-caspase 9, SLC7A11, GPX4, NRF2, p-EGFR, SirT2 and HRP-conjugated anti-rabbit IgG antibody were provided by Cell Signaling Technology (Boston, MA, United States). 5,5,6,6'-tetrachloro-1,1',3,3' -tetraethyl-imidacarbocyanine iodide (JC-1), 2',7'-Dichlorodihydrofluorescein diacetate (DCFH-DA), Cell Counting Kit-8 (CCK-8), Calcein-AM/PI double staining kit and Annexin V-FITC/PI Kit were obtained from Beyotime Biological Technology Co. Ltd. (Shanghai, China). BODIPY™ 581/591 C11 was purchased from Thermo Fisher Scientific (Waltham, MA). Other regents were purchased from Sigma-Aldrich (St. Louis, MO, USA).

### Cell lines and culture

The Human non-small cell lung cancer cell lines PC9, NCI-H1975, NCI-H1299, A549, NCI-H1650 and NCI-H8827 cells, and Human normal lung bronchial epithelial cells NHBE and BEAS-2B cells were obtained from American Type Culture Collection (ATCC, Manassas, VA, USA). The cells were maintained in RIPM 1640 Medium supplemented with 10% foetal bovine serum (FBS) and 1% penicillin/streptomycin solution. The cells were grown at an incubator containing 5% CO_2_ at 37 °C. The cells have no cross contamination of other human cell lines using the STR Multi-Amplification Kit (Microreader 21 ID System).

### High through-put screen

PC-9 cells were seeded at 96-well plate with a density of 5 × 10^3^ cells per well and cultured overnight. The cells were treated with compounds belonging to the Bioactive Compound Library purchased from Topscience, Inc (Shang hai) at the concentration of 10 µM. And DMSO was set as control. After incubation for 48 h, 10 μL CCK-8 regents were added to each plate and incubated for another 3 h. The absorbance was measured using a SynergyMx Multi-Mode Microplate Reader (Biotek, Winooski, VT) at 450 nm.

### Cell proliferation assay

The effect of tenovin-3 on the PC9 cell proliferation was detected using CCK-8 kit according to the manufacturer’s instructions. Briefly, the cells were seeded into 96-well plates and exposed with various concentration of tenovin-3 for 48 h or 72 h. After that, 10 μL CCK-8 regents were added to each well and further incubated for 2 h. Finally, the absorbance was detected at 450 nm with a SynergyMx Multi-Mode Microplate Reader (Biotek, Winooski, VT).

### Calcein-AM/propidium Iodide (PI) staining assay

The Calcein-AM/PI double staining kit was used to quantify the number of living and dead cells. The PC9 cells were treated with various concentration of tenovin-3 for 48 h. After washing with PBS, the cells were stained with 2 µM Calcein-AM regent and 4.5 µM PI regent per well for 30 min at 37 °C. The living and dead cells were observed and photographed using an EVOS XL Core (Thermo Fisher Scientific).

### Colony formation

The PC9 cells were seeded in 6-well plate at a density of 1 × 10^3^ cells per well and treated with various concentration of tenovin-3 for 72 h. The culture medium was replaced twice a week. After 2 weeks, the survival colonies were stained with crystal violet solution for 15 min and photographed using an EVOS XL Core (Thermo Fisher Scientific). The number of colonies was calculated using with Image-Pro Plus 6.0 software (Media Cybernetics, Rockville, MD, USA).

### Flow cytometric analysis of apoptosis

The PC9 cells were plated in 6-well plate and treated with different concentration of tenovin-3 for 48 h. And the cells were collected, washed with cold PBS twice and stained with 300 µL binding buffer containing 1 µL propidium iodide (PI) and 2 µL Annexin V for 15 min at room temperature in the dark. After that, the cells were analyzed with a flow cytometer (Beckman Coulter, Inc.) and the apoptotic cells were calculated using FlowJo™10.6.2 Software (BD Life Sciences) (URL link: https://flowjo.bectondickinson.cn/).

### Western blotting

The cells with indicated treatment were collected and lysed in RIPA lysis buffer. Equal amount of protein was separated with 15% SDS-PAGE gels and subsequently transferred onto PVDF membranes. The membranes were blocked with 5% BSA and then incubated with different primary antibodies at 4 °C overnight, following with incubation of anti-rabbit and anti-mouse IgG HRP-conjugated antibodies at room temperature. Proteins bands were visualized with chemiluminescent (ECL) detection kit (Millipore, USA). β-tubulin or GAPDH was set as loading control. The blots densities were quantified by Image J.

### Measurement of total ROS

The level of ROS was evaluated with DCFH-DA using a laser scanning confocal microscope (LSM 800, ZEISS). Briefly, the PC9 cells that treated with or without different concentrations of tenovin-3 were incubated with 10 µM DCFH-DA for 0.5 h in dark. After washing with PBS, the cells were observed with a laser scanning confocal microscope at an excitation wavelength of 488 nm and an emission wavelength of 525 nm. And the Hoechst staining was used to recognize nuclear.

### Glutathione (GSH) detection assay

The production of GSH was detected using a GSH assay kit following with the manufacturer's instructions. Briefly, PC9 cells with indicated treatment were collected and dissolved in M solution. And then, the cells were frozen and thawed twice using liquid nitrogen and aqueous solution quickly. After incubating on the ice for 5 min, the GSH in the supernatant was obtained by centrifuging (10000 *g*, 10 min). As for the total GSH, the cells were diluted in M solution for 10 times. The concentration of GSH was measured with a stander curve following with the manufacturer’s instructions. The absorption was measured on a microplate reader at 412 nm.

### Determination of mitochondrial membrane potential (MMP)

The MMP alteration of PC9 cells after tenovin-3 treatment was detected by a JCI-1 staining kit according to manufacturer's instructions. In brief, PC9 cells with indicated treatment were stained with JC-1 reagent (2.5 µg/mL) for 30 min in the dark. Hoechst staining was used to nuclear staining. The cells were observed using a laser scanning confocal microscope. In living cells, JC-1 is a monomer in the cytosol (green fluorescence) and accumulates in the mitochondria (red fluorescence). In dead or dying cells, JC-1 do not accumulate in mitochondria due to the mitochondrial potential collapses.

### TEM assay

After treating with different concentration of tenovin-3 for 48 h, the PC9 cells were fixed with 2.5% glutaraldehyde dissolved in 0.1 mM sodium cacodylate buffer overnight at 4 °C. Subsequently, the cells were post-fixed in 1% osmium tetroxide and 0.1% potassium ferricyanide, followed by a graded series of ethanol. And then, the cells were embedded in epoxy resin and cut into ultrathin Sects. (60–80 nm) using an ultramicrotome (Leica EM UC7, Solms, Germany). The sections were stained with 2% uranyl acetate saturated alcohol solution and lead citrate. The images were obtained using a transmission electron microscope.

### Statistical analysis

All the results were analyzed using GraphPad Prism 5.0 (GraphPad Software, Inc., San Diego, CA, USA) and represented as the mean ± SD. The unpaired Student’s t test was used to analyze the significance between two groups. And One-way analysis of variance (ANOVA) followed by Tukey’s test were used for multiple comparisons. *P* < 0.05 was considered to indicate a statistically significant difference.

## Supplementary Information

Supplementary Information.

## Data Availability

The data presented in this study are available on request from the corresponding author.
