# Author Correction: Tenovin 3 induces apoptosis and ferroptosis in EGFR 19del non small cell lung cancer cells

**DOI:** 10.1038/s41598-024-68087-2

**Published:** 2024-07-24

**Authors:** Sha Lv, Qianrong Pan, Weijin Lu, Weisong Zhang, Naike Wang, Lijuan Huang, Lianjing Li, Jieyao Liu, Jiamei Ma, Zhan Li, Yong Huang, Qiudi Deng, Xueping Lei

**Affiliations:** 1 The Fifth Affiliated Hospital, Guangdong Province & NMPA & State Key Laboratory, School of Pharmaceutical Sciences, Guangzhou Medical University, Guangzhou, 511436 People’s Republic of China; 2 The Fifth Affiliated Hospital of Jinan University (Heyuan Shenhe People’s Hospital), Heyuan, 517000 China; 3 GMU-GIBH Joint School of Life Sciences, The Guangdong-Hong Kong-Macau Joint Laboratory for Cell Fate Regulation and Diseases, Guangzhou Medical University, Guangzhou, 511436 People’s Republic of China; 4 Medicine and Health Science College, Guangzhou Huashang College, Guangzhou, People’s Republic of China

Correction to: *Scientific Reports* 10.1038/s41598-024-58499-5, published online 01 April 2024

The original version of this Article contained an error in the spelling of the author Weijin Lu which was incorrectly given as Weijing Lu.

The original Article has been corrected.

