# Correction: Tenovin 3 induces apoptosis and ferroptosis in EGFR 19del non small cell lung cancer cells

**DOI:** 10.1038/s41598-025-30191-2

**Published:** 2025-12-03

**Authors:** Sha Lv, Qianrong Pan, Weijin Lu, Weisong Zhang, Naike Wang, Lijuan Huang, Lianjing Li, Jieyao Liu, Jiamei Ma, Zhan Li, Yong Huang, Qiudi Deng, Xueping Lei

**Affiliations:** 1 The Fifth Affiliated Hospital, Guangdong Province & NMPA & State Key Laboratory, School of Pharmaceutical Sciences, Guangzhou Medical University, Guangzhou, 511436 People’s Republic of China; 2 The Fifth Affiliated Hospital of Jinan University (Heyuan Shenhe People’s Hospital), Heyuan, 517000 China; 3 GMU-GIBH Joint School of Life Sciences, The Guangdong-Hong Kong-Macau Joint Laboratory for Cell Fate Regulation and Diseases, Guangzhou Medical University, Guangzhou, 511436 People’s Republic of China; 4 Medicine and Health Science College, Guangzhou Huashang College, Guangzhou, People’s Republic of China

Correction to: *Scientific Reports* 10.1038/s41598-024-58499-5, published online 01 April 2024

The original version of this Article contained errors in Figure 2.

Due to an error during figure assembly, the image for Calcein-AM/ PI staining assay’ in 2 μM Tenovin-3group in Figure 2B was a duplication of the image for 4 μM Tenovin-3group in Figure 1F.

In addition, in Figure 2E, two GAPDH blots were given instead of a single GAPDH loading control.

The original Figure 2 and accompanying legend appears below.

**Fig. 2:**
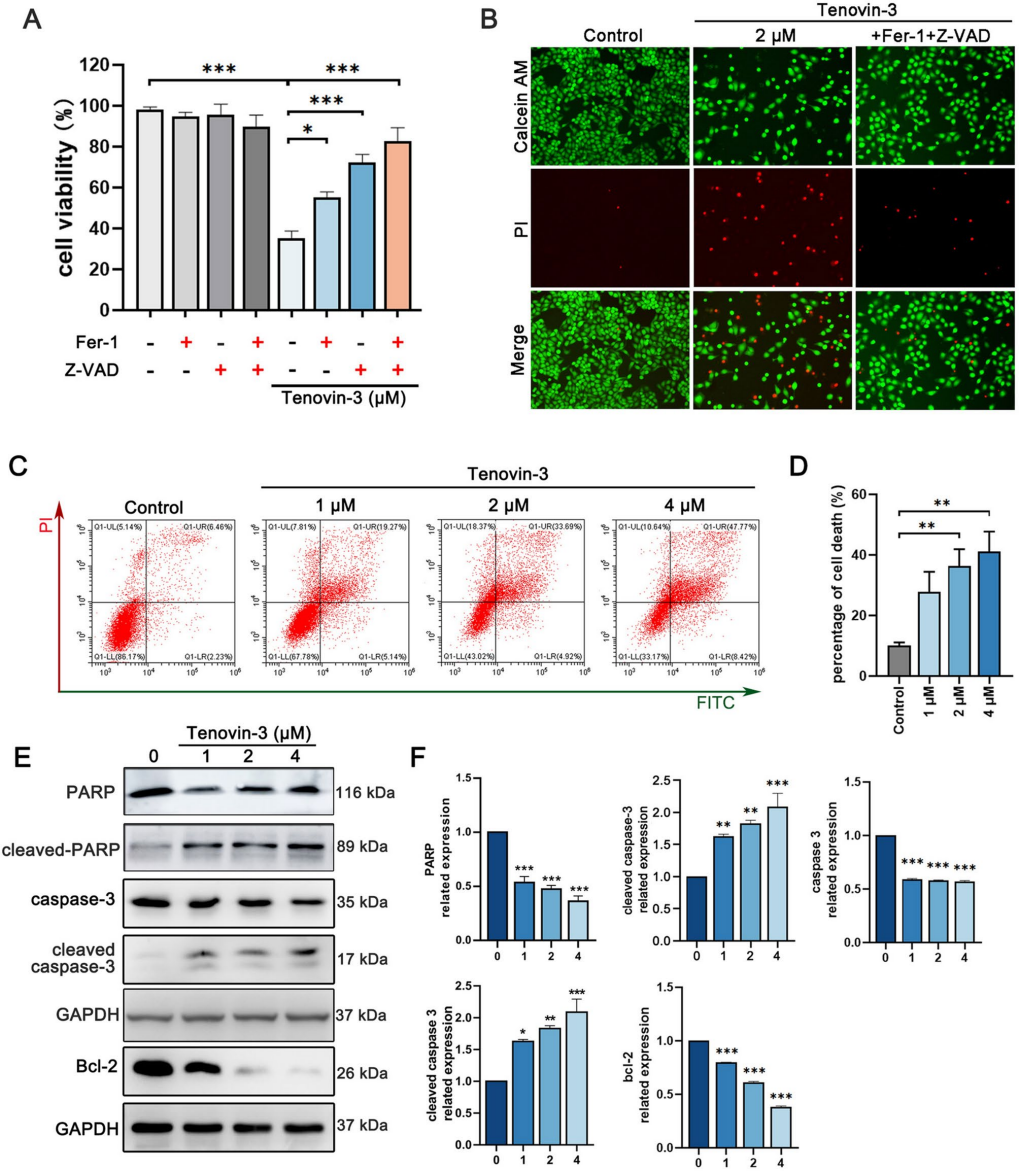
Tenovin-3 induces PC9 cells apoptosis. (**A,B**) The PC9 cells were co-treated with tenovin-3, apoptosis inhibitor Z-VAD-FMK or ferroptosis inhibitor Fer-1, and the cell viability was detected by CCK-8 assay (A) and Calcein-AM/ PI staining assay (**B**). (**C,D**) Tenovin-3 induced PC9 cells apoptosis indicated by flow cytometer assay. PC9 cells were exposure to various concentrations of tenovin-3 for 48 h, and then Annexin V/PI assay was used to detect apoptosis rate. The representative images and statistical data were displayed in (**C**) and (**D**). (**E–F**) The effect of tenovin-3 on the expression of apoptosis related protein detected by Western blotting. PC9 cells were treated with various concentrations of tenovin-3, and the cells were collected and subjected for Western blotting. GAPDH was set as a loading control. The representative blots and statistical data were presented in (**C**) and (**D**). The blots were cut and then incubated with antibodies. The uncropped version of the western blots is displayed in Supplementary Fig. 8. The data are presented as mean ± SD, n = 3. *P < 0.05, **P < 0.01, ***P < 0.001 compared with the control group.

The original Article has been corrected.

