# Supplementary material for: Tenovin 3 induces apoptosis and ferroptosis in EGFR 19del non small cell lung cancer cells
Source: Sci Rep. 2024 Apr 1;14:7654. doi: 10.1038/s41598-024-58499-5 (PMC10985106; doi:10.1038/s41598-024-58499-5)

**Supplementary Information for**

**Tenovin 3 induces apoptosis and ferroptosis in EGFR 19del non small cell lung cancer cells**

Sha Lv^a, 1^, Qianrong Pan^a, 1^, Weijing Lu^b, 1^, Weisong Zhang^a^, Naike Wang^c^, Lijuan Huang^a^, Lianjing Li^a^, Jieyao Liu^a^, Jiamei Ma^d^, Zhan Li^a^, Yong Huang^a^, Qiudi Deng^c, *^, Xueping Lei^a, *^

^a^ Guangzhou Municipal and Guangdong Provincial Key Laboratory of Molecular Target & Clinical Pharmacology, the NMPA and State Key Laboratory of Respiratory Disease, School of Pharmaceutical Sciences & The Fifth Affiliated Hospital and The sixth Affiliated Hospital, Guangzhou Medical University, Guangzhou, 511436, PR China

^b^ Shenhe People's Hospital (The Fifth Affiliated Hospital of Jinan University), Heyuan 517475, China

^c^ GMU-GIBH Joint School of Life Sciences, The Guangdong-Hong Kong-Macau Joint Laboratory for Cell Fate Regulation and Diseases, Guangzhou Medical University, Guangzhou, 511436, PR, China

^d^ Medicine And health science college, Guangzhou Huashang College

^*^Correspondence to: xuepinglei@gzhmu.edu.cn (Xueping Lei), dengqiudi@gzhmu.edu.cn (Qiudi Deng).

^1^ These authors contributed equally to this work

**Supplementary Table 1.** **The information of compounds that Z-score>4 in high throughput screening.**

| Compound | CAS |
| --- | --- |
| Salidroside | 10338-51-9 |
| (+)-JQ-1 | 1268524-70-4 |
| Diflunisal | 22494-42-4 |
| Docetaxel | 114977-28-5 |
| HTH-01-015 | 1613724-42-7 |
| Theophylline-7-acetic acid | 652-37-9 |
| AMI-1 | 20324-87-2 |
| Iniparib | 160003-66-7 |
| C7280948 | 587850-67-7 |
| EPZ011989 | 1598383-40-4 |
| Curcumol | 4871-97-0 |
| AZD1208 | 1204144-28-4 |
| J147 | 1146963-51-0 |
| SMI-4a | 438190-29-5 |
| UNC1999 | 1431612-23-5 |
| HPOB | 1429651-50-2 |
| Tenovin-3 | 1011301-27-1 |
| CID-2011756 | 638156-11-3 |

**
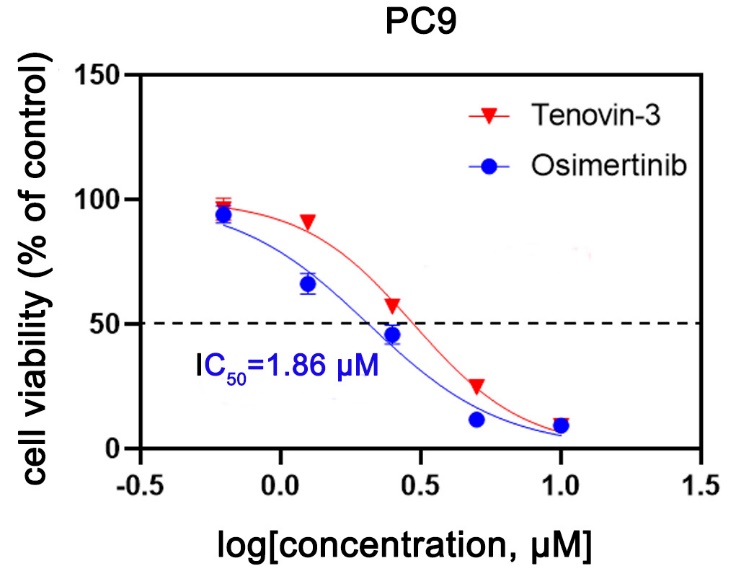
**

**Supplementary** **Fig. 1. The** **effect of tenovin-3 and Osimertinib on PC9 cells proliferation.** PC9 cells were treated with different concentration of tenovin-3 or Osimertinib for 48 h. And the cell viabilities were detected with CCK-8 assay.

**
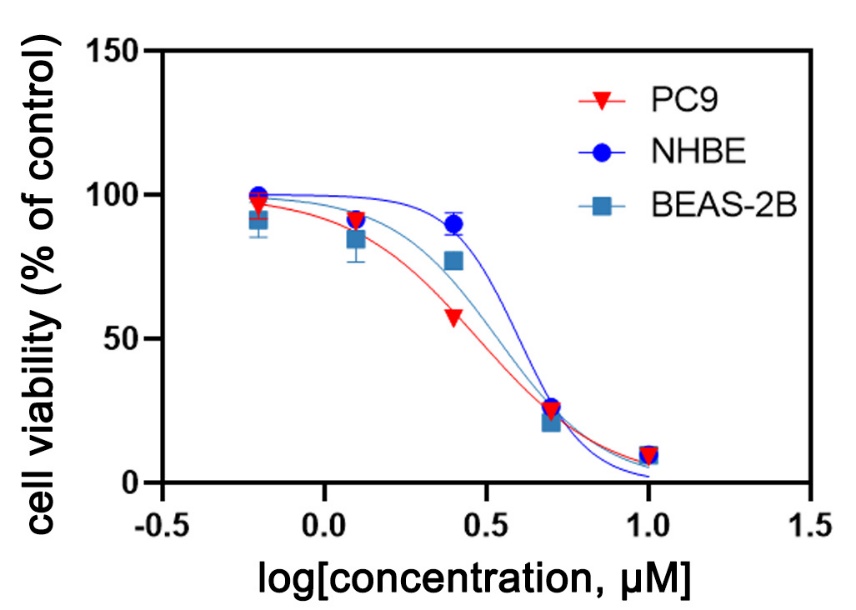
**

**Supplementary Fig.2. The effect of tenovin-3 on NHBE and BEAS-2B cells. P**C9, NHBE and BEAS-2B cells were treated with different concentration of tenovin-3 for 48 h. And the cell viabilities were detected with CCK-8 assay.


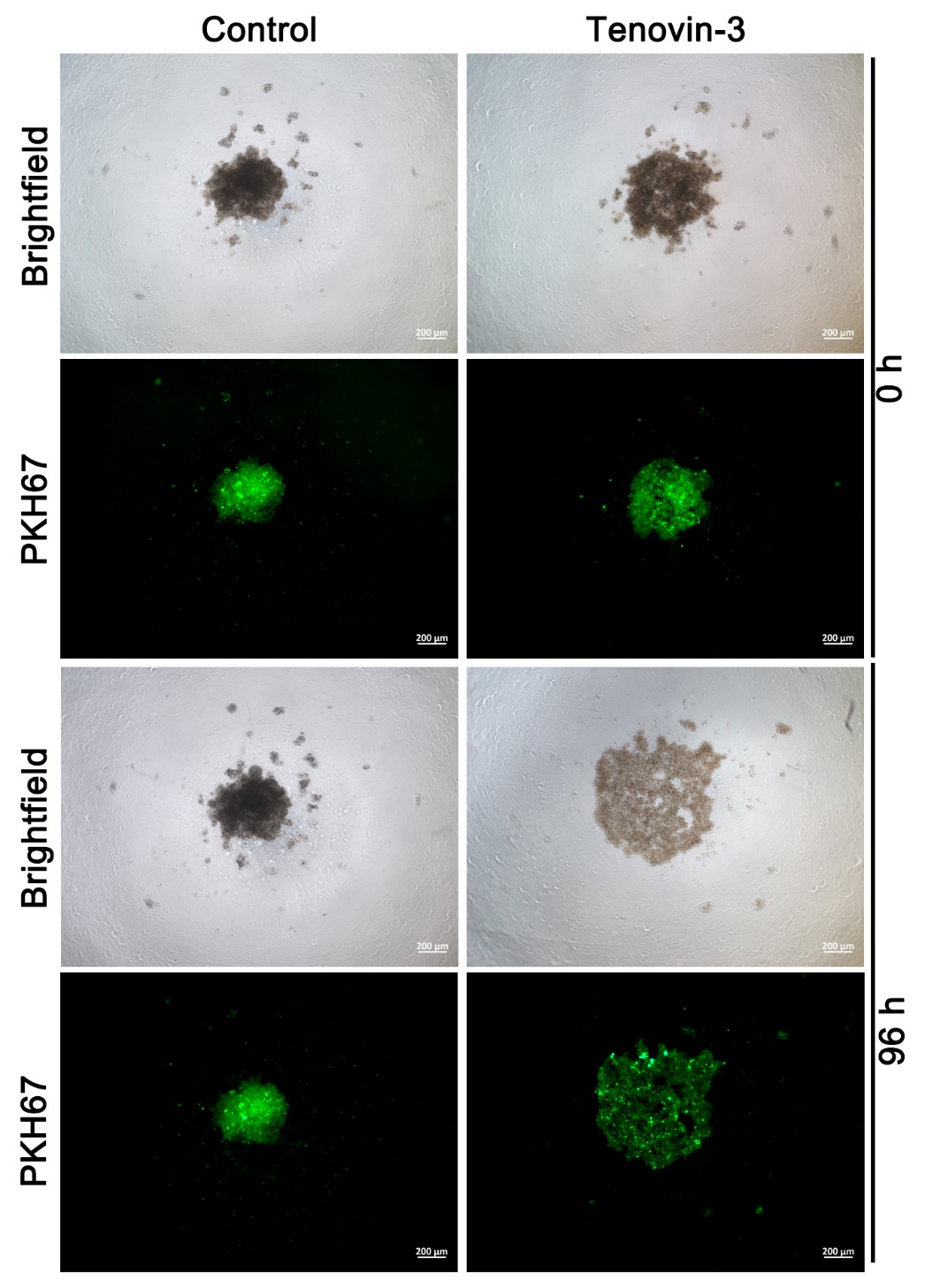


**Supplementary Fig.3. The anti-tumor effect of tenovin-3 detected by 3D spheroid assay.** The PC9 cells were labeled with PKH67 (green), and then were co-cultured with cancer associated fibroblasts in spheroid microplates plates (Corning) for 72h to allow the cells form spheroids. Then, the spheroids were treated with or without for tenovin-3 for 48 h. After cultured fresh medium for another 48 h, the spheroids were observed and photographed. And the time when tenovin-3 was added were set as 0 h.

**
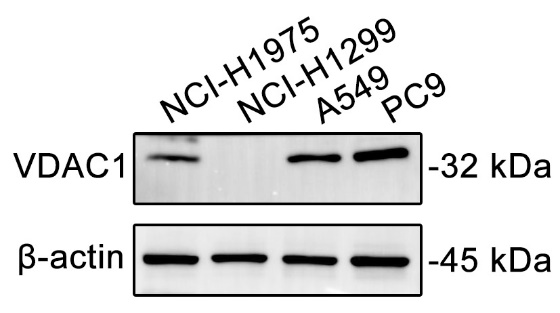
**

**Supplementary Fig. 4. The VDAC level in PC9, NCI-H1299, A549 and NCI-H1975 cells.** The blots were cut prior to incubation with antibodies. The uncropped version of the western blots is presented in Supplementary Fig.8.


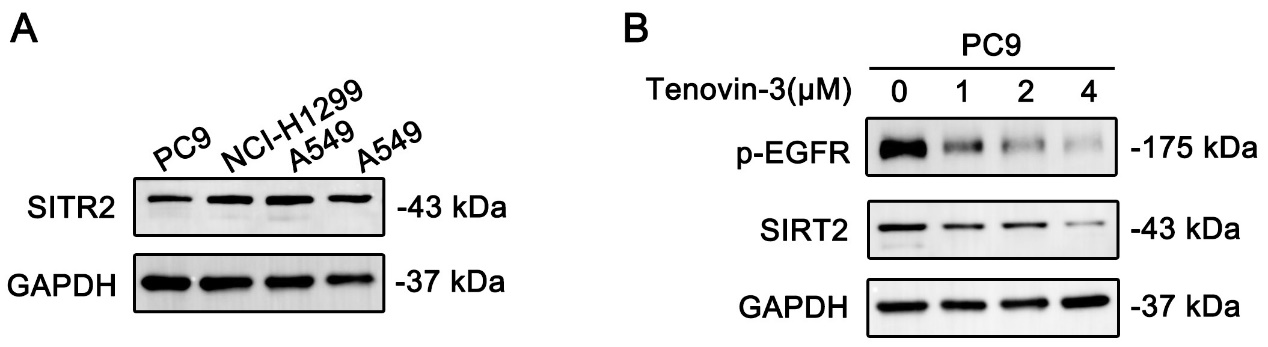


**Supplementary Fig. 5.** **The effect of tenovin-3 on p-EGFR and SITR2 expression.** (A) The SITR2 expression in PC9, NCI-H1299, A549 and NCI-H1975 cells. (B) The effect of tenovin-3 on p-EGFR and SITR2 expression. The uncropped version of the western blots is presented in Supplementary Fig.8.


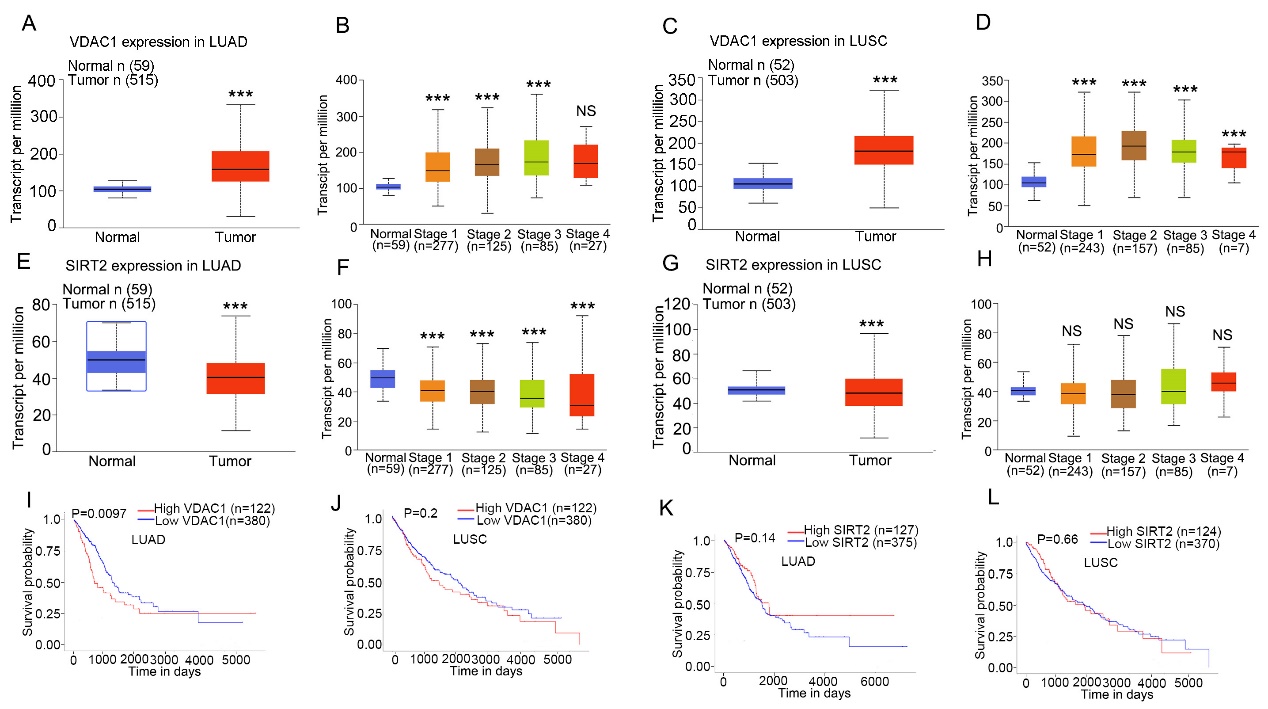


**Supplementary Fig. 6.** **The relationship of VDAC1 and SIRT2 expressions with LUAD and LUSC patients based TCGA platform. (A)** The VDAC1 expression is increased in LUAD tissues. **(B)** VDAC1 level is correlated with the individual cancer stages of LUAD. **(C-D)** The VDAC1 expression in LUSC tissues and its correlation with individual cancer stages of LUSC. **(E-F)** The SIRT2 expression in LUAD (**E**) and its expression is related with individual cancer stage (**F**). **(G-H)** The SIRT2 level in LUSC tissues (**G**) and it is correlated with individual cancer stage (**H**). **(I)** LUAD patients with high VDAC1 level had a better survival rate than that with low VDAC1 expression. **(J)** There is no significant correlation between VDAC1 expression and survival of LUSC patient. **(K-L)** There is no significant correlation between SIRT2 expression and survival of LUAD and LUSC patients. ^***^*P* < 0.001 compared with the normal tissues.

**
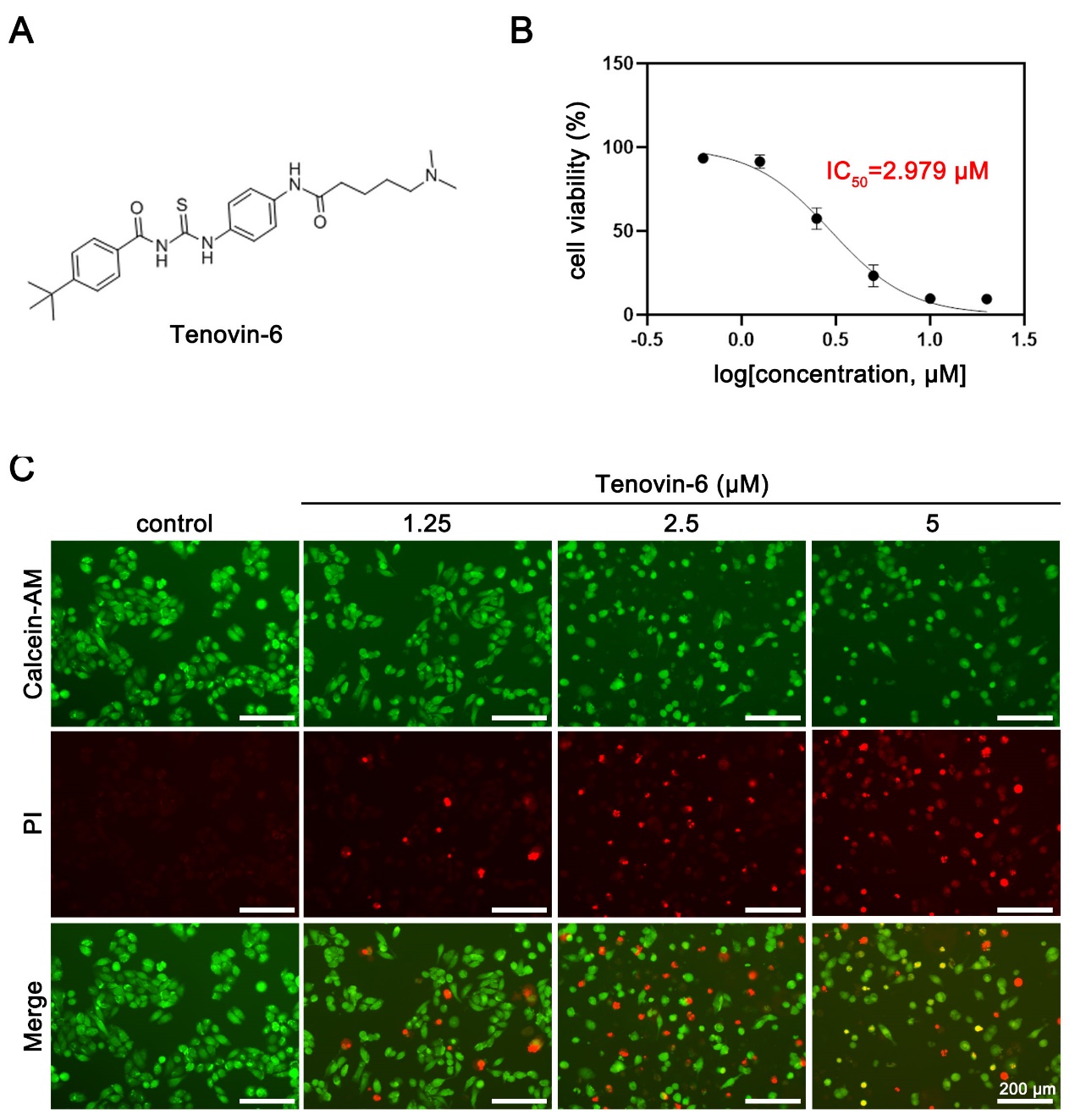
**

**Supplementary Fig. 7. Tenovin-6 suppresses the proliferation of PC9 cells. (A)** The chemical structure of Tenovin-6. **(B-C)** The effect of tenovin-6 on the proliferation of PC9 cells. The PC9 cells were treated with various concentration of tenovin-6, and the cell viability was detected by CCK-8 assay and Calcein-AM/ PI staining assay.

**Supplementary Fig. 8. The raw blots used in this study.**

**Figure 2E**

PARP


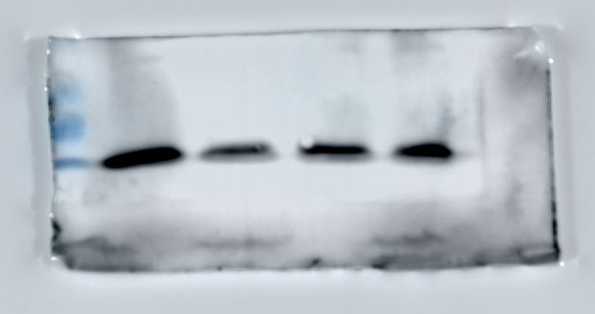


cleaved-PARP


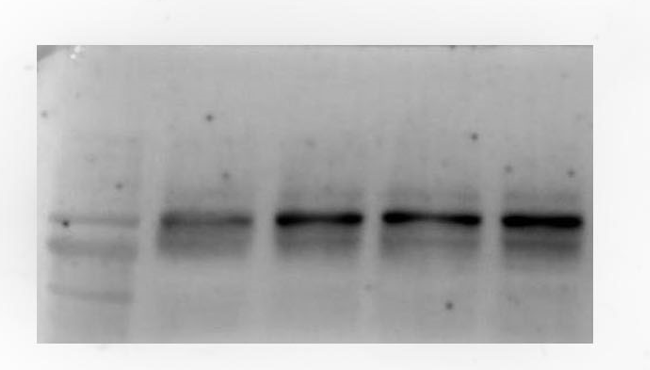


Caspase 3


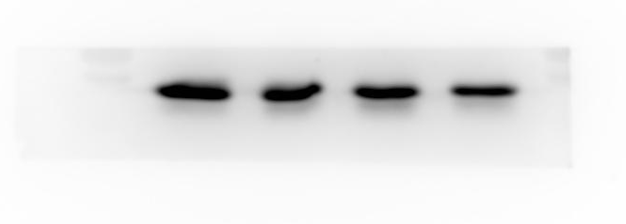


cleaved-Caspase 3


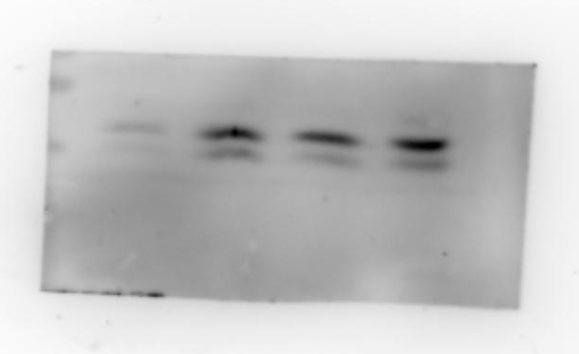


Bcl-2 Left 4 blots


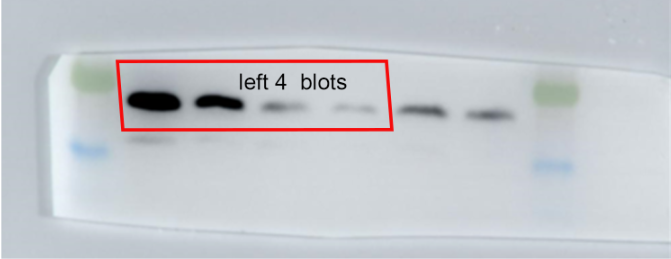


GAPDH


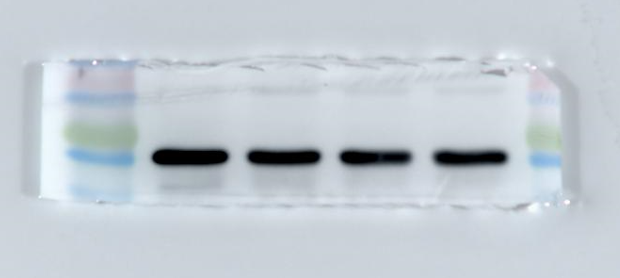


**Figure 3D**

NCOA4


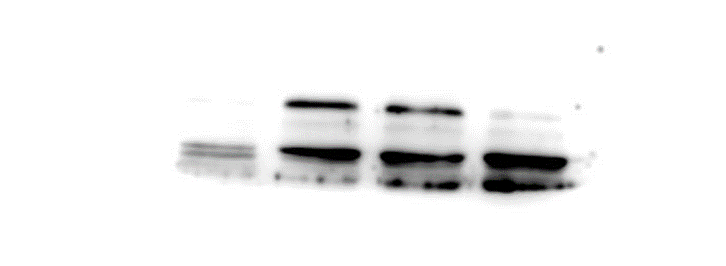


NRF2


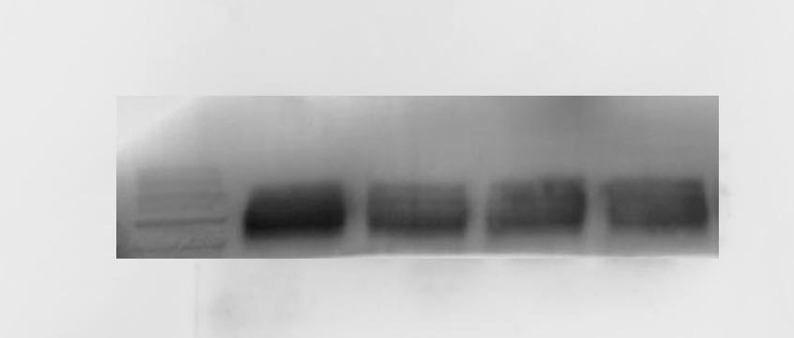


SLC7A11 left 4 blots


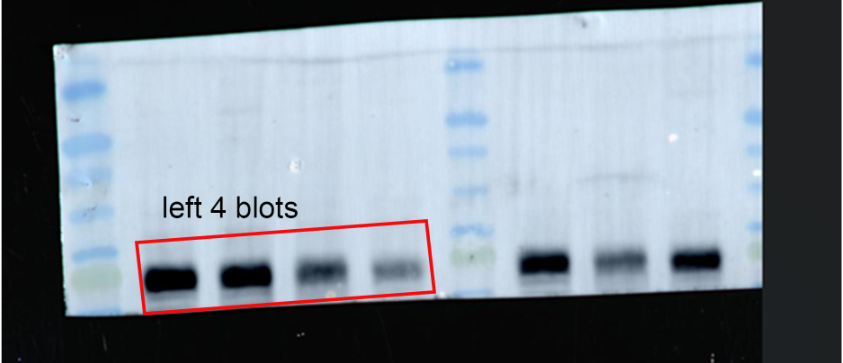


GPX4


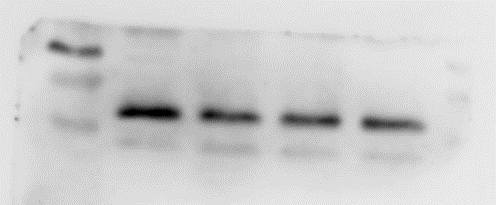


GAPDH


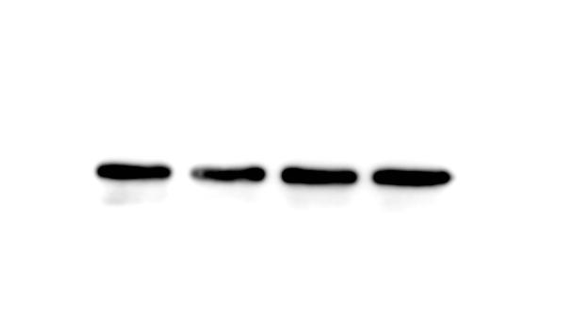


**Figure 4A**

VDAC left 4 blots


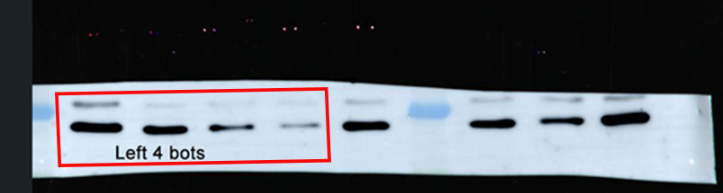


Cyt-c left 4 blots


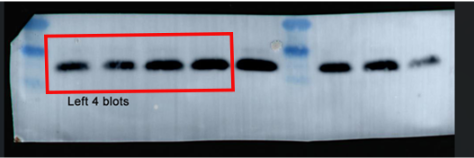


GAPDH


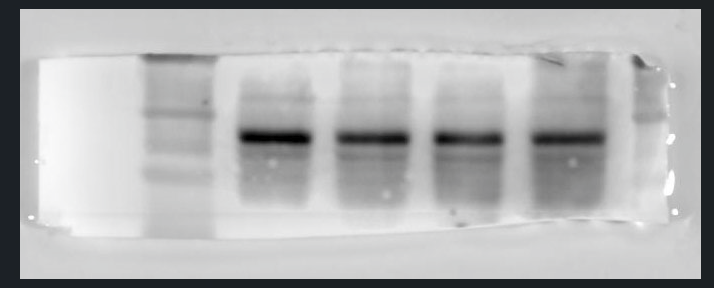


**Figure 4C**

VDAC Right 3 blots


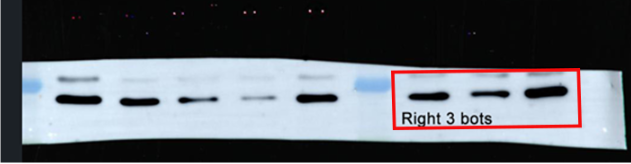


Cyt-c Right 3 blots


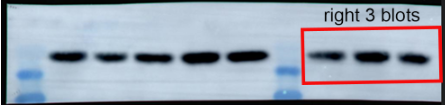


GAPDH Right 3 blots


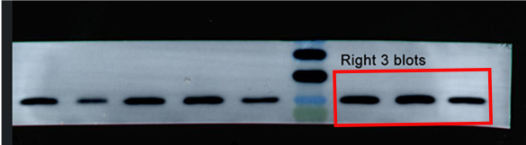


**Figure 4I**

PARP and cleaved PARP Right 3 blots


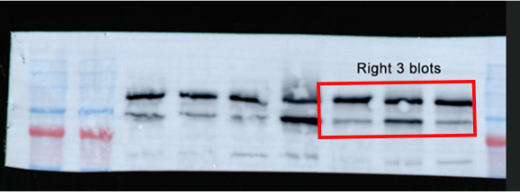


Cleaved Caspase 3 Right 3 blots


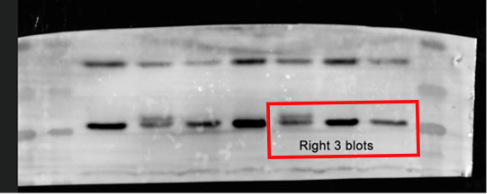


GAPDH Right 3 blots


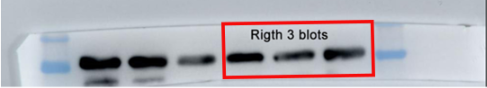


**Figure 5C**

SLC7A11 Right 3 blots


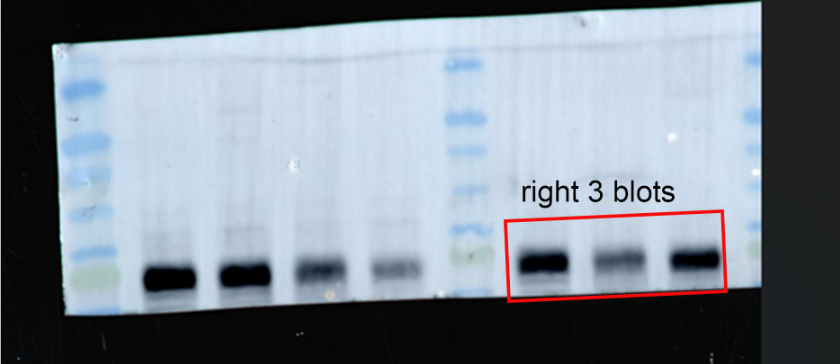


GPX4 Right 2-4 blots


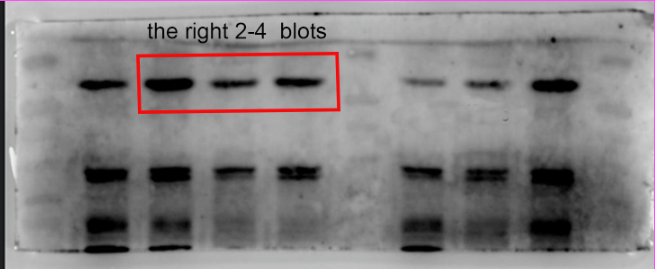


NRF2 The 2-4 blots


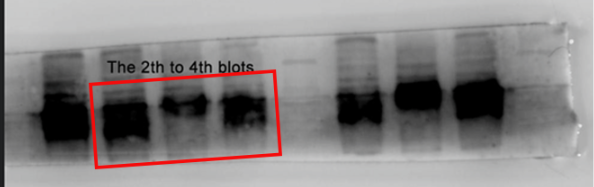


GAPDH


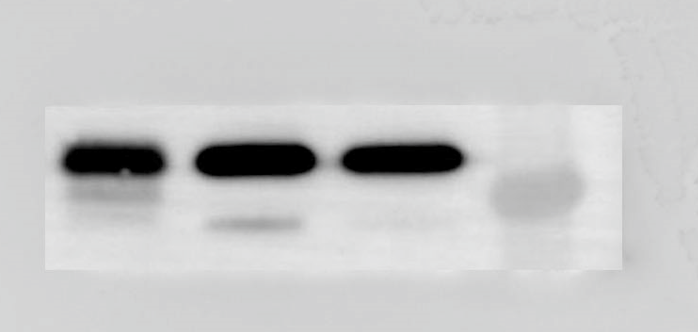


**Supporting Figure 1**

VDAC1 Right 4 blots


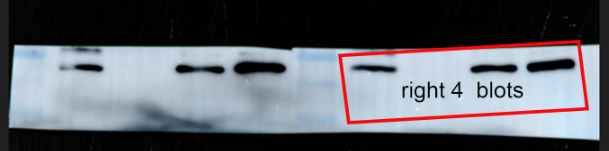


GAPDH Right 4 blots


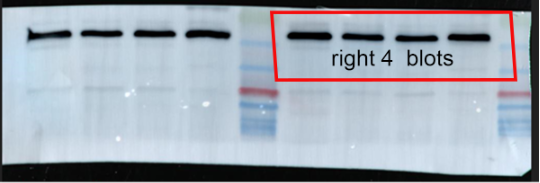


Supporting Fig.3 A

SITR2 Left 4 blots


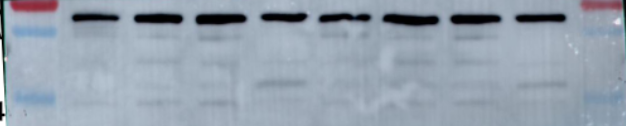


GAPDH Left 4 blots


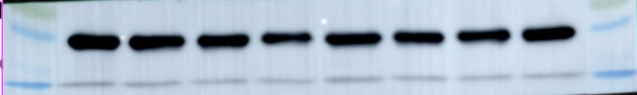


Supporting Fig.3 B

p-EGFR Right 4 blots


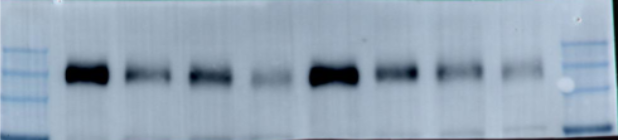


SIRT2 Right 4 blots


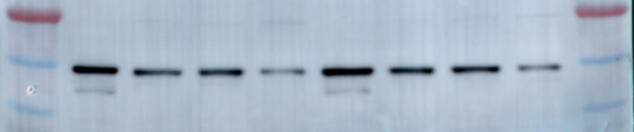


GAPDH Right 4 blots


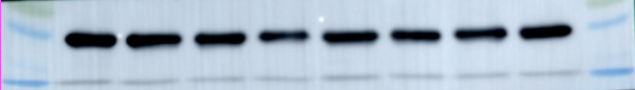

Supplement: Supplementary file 1 — Supplementary Information. [file 41598_2024_58499_MOESM1_ESM.docx]
